# The Biological Effects of Magnesium-Based Implants on the Skeleton and Their Clinical Implications in Orthopedic Trauma Surgery

**DOI:** 10.34133/bmr.0122

**Published:** 2024-12-23

**Authors:** Elena Müller, Till Schoberwalter, Konrad Mader, Jan-Marten Seitz, Alexander Kopp, Anke Baranowsky, Johannes Keller

**Affiliations:** ^1^ Department of Trauma and Orthopedic Surgery, University Medical Center Hamburg-Eppendorf, 20251 Hamburg, Germany.; ^2^ Medical Magnesium GmbH, 52068 Aachen, Germany.; ^3^ Meotec GmbH, 52068 Aachen, Germany.

## Abstract

Magnesium (Mg)-based implants have evolved as a promising innovation in orthopedic trauma surgery, with the potential to revolutionize the treatment of bone diseases, including osteoporotic fractures and bone defects. Available clinical studies mostly show excellent patient outcomes of resorbable Mg-based implants, without the need for subsequent implant removal. However, the occurrence of radiolucent zones around Mg-based implants seems to be a noticeable drawback for a more widespread clinical use. Mechanistically, both in vivo and in vitro studies demonstrated beneficial effects on the formation of new bone, a unique characteristic of Mg-based implants. In this regard, Mg has been shown to exert pleiotropic functions on osteogenic differentiation and migration of osteoblasts and their precursors. Additionally, collective evidence suggests that Mg-based implants promote angiogenesis in newly formed bone and exert immunomodulatory effects in the bone microenvironment. Likewise, Mg-based implants and their degradation products were shown to inhibit bone resorption by impairing osteoclastogenesis. The purpose of this review is to provide a state-of-the-art summary of the clinical and basic science evidence regarding the performance of currently used Mg-based implants. In addition to the status of in vivo and in vitro research and clinical applications, future challenges and perspectives of Mg-based orthopedic implants are discussed.

## Introduction

The development and optimization of bone fixation methods in the past decades has revolutionized the treatment of patients with skeletal injuries. Currently, the vast majority of available implants are made of nondegradable metals, like stainless steel, titanium and its alloys, or cobalt–chromium alloys. Although these implants are characterized by an excellent biocompatibility, high wear resistance, and sufficient mechanical strength [1], they nonetheless have critical limitations. Remaining metallic devices may produce difficulties in case of revision surgery. Furthermore, the high Young’s modulus of current metals may lead to peri-implant bone loss and even peri-implant fractures, often resulting in revision surgery [2]. Implant removal is not only required in case of complications caused by broken implants, pain, or immobility, but also at skeletal sites with thin soft tissue coverage such as the clavicula or distal fibula in which implants may trigger skin irritation. Overall, additional yuhsurgeries frequently cause significant comorbidities, including increased pain, prolonged and repetitive immobilization, muscle wasting, and decreased quality of life, in addition to high medical expenses [3]. Moreover, the traditional metals used in implants may interfere with the diagnostic accuracy of conventional radiographs and particularly computed tomography (CT) images due to beam hardening and imaging artifacts [4].

As potential alternative implant materials, natural and synthetic polymers have been developed to be applied in low weight-bearing skeletal sites [5,6]. Synthetic polymers include ultra-high molecular weight polyethylene (UHMWPE), poly(methylmethacrylate) (PMMA), polyurethanes (PUs), and polyetheretherketone (PEEK). As an advantage, these polymers do not cause radiographic artifacts and, due to the avoidance of stress shielding effects, have a low re-fracture risk. But potential toxic residual monomers and wear debris, in addition to the limited mechanical stability of synthetic polymers, increase clinical concerns and make them unsuitable for a wide range of orthopedic indications [7]. Natural polymers include collagen, chitosan, gelatin, silk fibroin, alginate, cellulose, and starch, alone or in combination. A high biocompatibility, an excellent biodegradability, and no toxicity are the main advantages [8]. However, there still exist some major challenges before a regular clinical use is possible. First, the fabrication of the scaffolds in a tolerable size, shape, and surface is challenging and new technologies are required, with bioprinting showing first promising results. Second, the vascularization of the large implants and their integration with the host tissues remain difficult [9].

Biodegradable Mg-based materials have emerged as an encouraging alternative to traditional titanium- or steel-based implants in orthopedic trauma surgery. These implants offer several advantages, including mechanical strength properties that closely mimic those of natural bone tissue and thus reduce a potential stress shielding situation in load-bearing applications [10,11]. In addition, being bioabsorbable, there is no need for a second surgery to remove the implant. Furthermore, and of utmost importance, Mg-based implants have been shown to induce osteogenesis, making them an attractive option for promoting bone formation and regeneration [12]. Several studies have highlighted the favorable effects of Mg ions derived from the insertion of Mg-based implants on bone formation and the tissue environment, including the promotion of angiogenesis, immunomodulatory and anti-inflammatory effects, and enhanced cell migration and adhesion [12,13]. The stimulatory effect of Mg ions derived from the insertion of Mg-based implants on the secretion of calcitonin gene-related peptide (CGRP), a neuropeptide that promotes osteogenesis, has been proposed as the reason for the increase in new bone formation [14].

Despite these potential benefits, the occurrence of radiolucent zones around the implant remains a noticeable drawback of Mg-based implants, potentially compromising bone integrity [15]. The exact nature and clinical implications of this phenomenon is not well understood, and further research is required to fully comprehend the benefits and drawbacks of Mg-based implants.

This review summarizes the state-of-the-art knowledge of the clinical and basic science evidence regarding the performance of currently used Mg-based implants. Available clinical studies employing Mg-based implants and their impact on the fractured and unfractured skeleton are discussed. These findings are benchmarked against experimental in vivo and in vitro studies, investigating the molecular bases of the observed effects. Finally, future challenges and perspectives of Mg-based orthopedic implants are also presented.

## Human Mg Physiology and Its Role in Bone Turnover

Mg (=Mg^2+^) is a chemical element with the atomic number 12. It is metal with low density, low melting point, and high chemical reactivity. Mg occurs in 3 stable isotopes: ^24^Mg, ^25^Mg, and ^26^Mg, with ^24^Mg being the most common isotope (78.99%). It has a melting point of 648.8 °C and a boiling point of 1,090 °C [16]. Mg is involved in practically every major metabolic and biochemical process within the cell [17]. For example, it serves for over 600 enzymes as cofactor, and for additional 200 as an activator [18,19]. Many of the enzymes that require Mg as coactivator are vital for life [17]. Mg has a hydrated radius, which is 400 times larger than its dehydrated radius. Consequently, it needs to be dehydrated before passing through channels and transporters, requiring a lot of energy [17]. Furthermore, Mg is a physiological calcium (Ca^2+^) antagonist and an important factor in the control of cell proliferation [17].

**Fig. 1.**
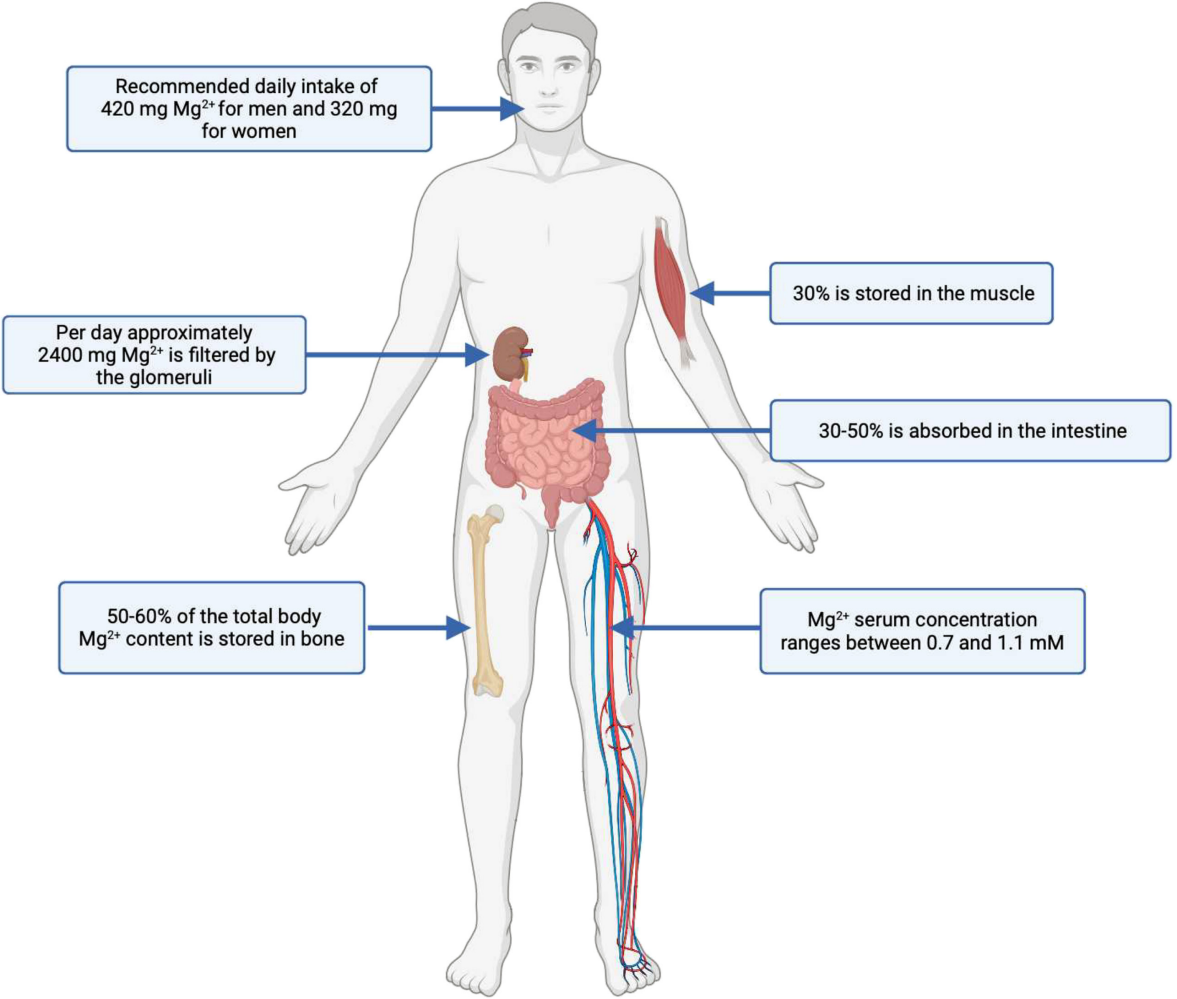
Schematic illustration of the distribution of Mg (Mg^2+^) throughout the human organism and its primary storage locations. Artwork created with Biorender.com.

Most of the body’s Mg is stored in bone, muscle, and soft tissues (Fig. 1). Only 1% of the body Mg content is in blood serum [17]. In healthy people, Mg serum concentration ranges between 0.7 and 1.1 mM [20]. A daily intake of 420 mg of Mg for men and 320 mg for women is recommended [21]. Mg  homeostasis bases on the interaction of the intestine (Mg uptake from food), the bone (storing Mg), and the kidneys (urinary excretion) [17]. Given a regular daily Mg intake, 30% to 50% is absorbed in the intestine (±100 mg), potentially increased up to 80% in case of a low intake [22]. About 50 to 60% of the total body Mg content is stored in bone [17]. Because bone surface Mg is continuously exchanged with blood Mg, serum concentrations are closely related to bone metabolism [23]. In bone, Mg ions bind at the surface of the hydroxyapatite crystals [17], and since Mg increases the solubility of P_i_ and Ca^2+^ hydroxyapatite, it affects crystal size and formation [24]. Additionally, Mg induces osteoblast proliferation. Mg deficiency increases the secretion of pro-inflammatory cytokines [e.g., tumor necrosis factor α (TNFα), interleukin 1 (IL1), and substance P [25,26]], all of which have been implicated in increased osteoclastic bone resorption [17,27]. Approximately 2,400 mg of Mg is filtered by the glomeruli every day. The nephron recovers 95 to 99% of this; the remaining Mg ions are excreted via urine [17]. Mg deficiency is associated with a large variety of diseases, inter alia neurological diseases such as migraine, depression, stroke, and epilepsy [17]. Low serum Mg levels are also associated with an increased risk of coronary artery disease, atherosclerosis, metabolic syndrome, and an increased risk of acute myocardial infarction and are frequently linked to high blood pressure [28–31]. Patients with type 2 diabetes mellitus or osteoporosis often show low serum Mg levels [32,33].

With respect to the skeleton, various studies have shown that hypomagnesaemia is associated with osteoporosis, especially in postmenopausal women [33,34]. Also, intervention studies have shown that supplementation with Mg in the form of citrate, carbonate, or oxide, with a dosage varying between 250 and 1,800 mg, yields an overall benefit in terms of both bone mineral density and fracture risk (discussed in detail elsewhere [35]). Apart from a direct effect on bone cells as discussed below, Mg deficiency may affect skeletal integrity indirectly by controlling the main regulators of Ca homeostasis, parathyroid hormone (PTH), and 1,25(OH)_2_ vitamin D_3_. Hypomagnesaemia was shown to inhibit the release of PTH and potentially reduce sensitivity to PTH signaling in target organs, thus mimicking hypoparathyroidism [36]. In turn, supplementation of Mg has been shown to normalize alterations in PTH and 1,25(OH)_2_ vitamin D_3_ in osteoporotic postmenopausal women [37]. Therefore, Mg exerts pleiotropic functions in the organism, both locally and systemically, which must also be taken into account in the case of Mg-based bioresorbable implants.

Most importantly, Mg positively affects bone regeneration by inducing new bone formation. In the search for the underlying mechanisms, studies have focused on the impact of Mg on osteogenesis, angiogenesis, and inflammation, as these 3 processes are essentially required for adequate bone healing. Collective evidence suggests that Mg-based implants stimulate bone formation not only by directly promoting osteogenesis of mesenchymal stem cells (MSCs) but also by the promotion of angiogenesis in the skeleton, in particular during bone regeneration. Also, some evidence is available that Mg may exert immunomodulatory effects in the bone microenvironment, with potential beneficial effect on bone regeneration.

## Origin and Published Clinical Trials of Mg-Based Implants in Orthopedic Surgery

Although the full potential of Mg-based implants has only been realized in recent decades, the first attempts to use Mg-based materials as medical implants go back a long way. The history of biodegradable Mg-based implants reaches back to the discovery of elemental Mg by Sir Humphrey Davy in 1808 [38,39]. In 1852, Robert Bunsen realized the commercial production of Mg by electrolysis [38]. In 1862, the first Mg products (for pyrotechnical use, and as igniting bands or wires for flash lights of the upcoming photographic industry) were presented at the world exhibition in London [38]. The first medical use can be dated to 1878. Back then, the physician Edward C. Huse documented the use of Mg wires to stop bleeding vessels in 3 cases [40]. In 1900, Payr wanted to use Mg plates and sheets in joint arthroplasties, but after frustrating animal experiments and first clinical observations, he abandoned this idea [41] and proposed Mg-based implants only as fixator pins, nails, wires (cerclage), pegs, cramps, sheets, and plates [42]. Furthermore, he recommended a Mg peg as an intramedullary stabilizer for irreducible bone fractures and nonunions [43]. In 1913, Groves published his experiments on rabbits, observing abscess cavities and a too quick dissolution to stabilize fractures with Mg-based implants [44]. Despite the critical published data, Verbrugge started in 1933 his in vivo research on Mg-based implants with a 2.5-mm-thick solid Mg cylinder in the femura of rabbits and dogs [45]. The Mg cylinders corroded slowly under moderate gas evolution, and Verbrugge declared that the concomitant gas evolution was not harming any tissue [45]. In his clinical investigation (21 cases), he observed hydrogen cavity formation without any adverse clinical effect [46].

Today, numerous case reports, case series, retrospective analysis, and prospective trials investigating the impact of Mg-based alloys in humans are available (Table 1).

**Table 1.** Summary of available clinical trials and case reports on Mg-based implants in orthopedic trauma surgery

Author	Type of study	Type of surgery	Implant	Number of patients	Follow-up	Major findings
Windhagen et al. [133]	Prospective study	Chevron osteotomy (hallux valgus)	MAGNEZIX CS 3.2	12	6 months	• Mg-based screw was radiographically and clinically equivalent to the conventional titanium screw
						• No foreign body reaction, osteolysis, or systemic inflammatory reaction
Plaass et al. [134]	Prospective study	Modified Chevron osteotomy (hallux valgus)	MAGNEZIX CS 3.2	40	21.4 weeks (6 weeks to 1 year)	• Mg-based implants degrade without an implant directed inflammation reaction and possess higher strengths than degradable polymer implants.
						• Proper training of implant handling is necessary
						• Different interindividual corrosion rates between patient loading conditions should be set with a safety margin to guarantee proper healing even in cases of early corrosion
Choo et al. [135]	Prospective study	Scarf osteotomy (hallux valgus)	MAGNEZIX CS 3.2	25	1 year	• Noninferiority of the MAGNEZIX implant when compared to titanium screws
						• No need for removal
						• No artifact when imaged with a CT scan
						• No concerns with the strength of the implant, implant prominence, or peri-implant fractures
						• Osteotomy healed uneventfully in all cases with no loss of metatarsal alignment
Yu et al. [136]	Retrospective study	Vascularized iliac grafting for displaced femoral neck fracture	4-mm pure Mg screws	19	16 months	• Satisfactory results with a low rate of complications including avascular necrosis and nonunion
Lee et al. [137]	Retrospective study	Fixation of hand fractures	Mg-5wt%Ca-1wt%Zn screw	53	1 year	• Complete bone healing
Plaass et al. [138]	Retrospective study, titanium vs. magnesium screws	Distal metatarsal osteotomies (hallux valgus)	MAGNEZIX CS 3.2	8	3 years	• No statistical relevant difference between Mg und titanium screws
						• Significantly less artifacts in the MRI
						• No implant related cysts, implant under degradation
Acar et al. [139]	Retrospective study, titanium vs. magnesium screws	Modified chevron osteotomy (hallux valgus)	MAGNEZIX CS 2.7	17	12 months	• Therapeutic efficacy and complication rates were seen to be comparable with titanium screw fixation
						• No implant removal operations
Kose et al. [140]	Retrospective study	Medial malleolar fracture fixation	MAGNEZIX CS 3.2	11	17.3 moths (12–24 months)	• Adequate fixation with good functional results.
						• AOFAS score 94.9 ± 5.7 points (85–100 points), mean VAS score 0.4 ± 1.2 points (0–4 points).
						• Radiographic solid union in all cases
						• No complications during the follow-up
Atkinson et al. [108]	Retrospective study, titanium vs. magnesium screws	Modified short scarf osteotomy (hallux valgus)	MAGNEZIX CS 3.2	11	19 months (12–30 months)	• No statistical relevant difference between magnesium and titanium screws
						• Adequate fixation with good patient-reported outcomes
Klauser [109]	Retrospective study, titanium vs. magnesium screws	Distal MT I osteotomy (hallux valgus)	MAGNEZIX CS 3.2	95	12.2 weeks	• Mg-based screws were statistically noninferior to the conventional titanium screws
						• No need to remove Mg screws
						• No significant differences in mechanical stability, wound healing, or infection rate
						• 40% implant-specific phenomena in postoperative radiographs
						• Complete consolidation in all cases
Acar et al. [141]	Retrospective study, titanium vs. magnesium screws	Medial malleolar osteotomy for osteochondral lesions of the talus	MAGNEZIX CS 3.2	11	1 year (12–49 months)	• Similar rates of union and functional outcomes
						• None of the patients had displacement or malunion
						• Unusual radiographic findings during the follow-up—neither caused any clinical symptom nor interfered with fracture union
Leonhardt et al. [142]	Retrospective study	Mandibular condyle fracture fixation	MAGNEZIX CS 2.7	6	12 months	• All patients showed well-restored function of the temporomandibular joint with significant improvement in mouth opening, right and left laterotrusion, and protrusion distances to physiologic values
Lee et al. [143]	Retrospective study	Fixation of Mason type II radial head fractures	2.7-mm headless screws (Resomet; U&I Corp., Seoul, Korea)	22	18.9 months (12–38 months)	• Satisfactory results in radiographic and clinical evaluations
						• Sufficient fixation force at the fracture site
Deichsel et al. [122]	Case report	Refixation fragment osteochondrosis dissecans knee	2 cannulated 2.8 × 20 mm headless magnesium compression screws (Medical Magnesium GmbH, Aachen, Germany)	1	14 months	• Pain-free (VAS-score 0 of 10) and no movement restrictions
Wichelhaus et al. [118]	Case report	Partial wrist fusion	MAGNEZIX headless bone screw	1	6 months	• Early degradation of the screws lead to mechanical instability resulting in nonunion and osteolysis of the 3 carpal bones
Biber et al. [110]	Case report	Distal fibula fracture fixation	MAGNEZIX CS 3.2	1	17 months	• Uneventful healing
						• Radiolucency, formed within 6 weeks postoperatively, disappeared after 17 months
Biber et al. [144]	Case report	Osteochondral fracture fixation humeral capitulum	MAGNEZIX CS 3.2	1	1 year	• Clinical course uneventful
						• Degradation of the magnesium alloy did not interfere with fracture healing
						• Excellent clinical result and free range-of-motion
Turan et al. [111]	Case report	Radial styloid fracture fixation	MAGNEZIX CS 2.7	2	48 months/6 months	• Mg screws provide compression, can be applied percutaneously, and implant removal operations are abandoned
						• Fracture union was achieved without any complication in both cases
Acar et al. [145]	Case reports	Distal fibula fracture fixation	MAGNEZIX CS 3.2	1	2 years	• No failure of the implant or re-displacement of the fracture
						• No interference of radiolucency with early phases of fracture union or final consolidation
Zhao et al. [146]	Case report	Fixation of vascularized bone graft in osteonecrosis of the femoral head	Pure Mg screw	1	2 years	• Anabolic effects of degraded Mg ions with greater bone formation around the screw and flap fusion region
						• Degradation rate was tolerable without observable local gas formation
						• No tissue necrosis around screw, no abnormal blood chemistry

Most published studies were performed on the Mg-based MAGNEZIX screws (Syntellix AG, Hannover, Germany) used in hand, foot, and ankle surgery. In addition to less or no artifact on magnetic resonance imaging (MRI) and CT, respectively, and no need for implant removal, the prospective studies consistently reported noninferiority to titanium screws. Likewise, these studies also described no implant-directed or systemic inflammatory responses, or foreign body reaction. Most available retrospective studies have shown similar results, with overall noninferiority to titanium in terms of mechanical stability and incidence of wound healing or postoperative infection, as well as good patient-reported outcomes. The occurrence of radiolucent zones around Mg-based implants has been described primarily in case reports, the vast majority of which conclude that this phenomenon does not interfere with fracture healing. However, there are also a few case reports that have described excessive formation of radiolucent zones, osteolysis, and even nonunion in patients treated with Mg-based screws, which is discussed in detail below.

Together, the available evidence from the above clinical studies on biomedical Mg ions derived from the insertion of Mg-based implants show overall consistent and encouraging results [3]. Apart from the main advantage of bioabsorbability, most studies have confirmed the osteoanabolic, i.e., osteoinductive, effect of Mg ions derived from the insertion of Mg-based implants on new bone formation. However, controlling the rapid degradation and high corrosion rates of degradable Mg alloys in the human body and, most importantly, understanding the nature of the associated radiolucent zones as discussed below remain the main barriers to more widespread clinical use of Mg-based implants [3]. Therefore, a better understanding of the underlying cellular and molecular responses of the organism toward Mg-based implants is needed to further allow optimization of designs and applications of Mg-based implants.

## Impact of Mg-Based Implants on Bone Regeneration and Formation

One of the most remarkable effects of bioresorbable Mg ions derived from the insertion of Mg-based implants is the induction of new bone formation. Since only a few osteoanabolic agents, including systemic injections of intermittent teriparatide and topical application of bone morphogenetic proteins (mostly BMP2), are clinically available to promote bone repair, this property of Mg ions derived from the insertion of Mg-based implants has attracted considerable attention and prompted numerous studies to investigate the underlying mechanisms. To date, a wide range of experimental studies have shown that Mg-based implants have a superior effect on fracture healing compared to conventional implants [12,14,47–49] (Table 2).

**Table 2.** Summary of available in vivo and in vitro studies on the biological effects of Mg in skeletal tissues

Author	Type of study	Type of surgery/animal	Implant	Control	Major findings
Ye et al. [48]	In vivo/in vitro	Distraction osteogenesis in a rat model of a critical size mid-shaft femoral defect	Intramedullary Mg nail implantation	Pure distraction osteogenesis	• Mg group: higher CGRP expression in the periosteum and increased angiogenesis in vivo
					• Mg increases angiogenesis in vivo through CGRP signaling
Han et al. [47]	In vivo/in vitro	Rabbit femoral intracondylar fracture fixation model	High-purity Mg screws (99.99 wt % Mg)	PLLA screws	• Mg screws: improved osseointegration; higher bone volume and bone mineral density around the screws; bone-to-implant contact area increased significantly; accelerated bone formation in fracture gap
					• High concentrations of Mg ions (3.25 mM Mg, Mg screw extract) in human BMSC cultures resulted in significantly increased cell viability compared to controls
					• Alkaline phosphatase activity and mRNA levels of osteoblastic markers *osteopontin*, *runt-related transcription factor 2*, and *alkaline phosphatase* significantly higher after 14 d, suggesting enhanced osteoblastic differentiation
Zhang et al. [14]	In vivo/in vitro	Closed femoral fracture model in rats with ovariectomy-induced osteoporosis	Mg rod inserted into a hollow porous stainless-steel intramedullary nail (Mg-IMN)	normal IMN	• Accelerated healing
					• Mg-IMN group performed significantly better in a 4-point bending biomechanical test at week 12
					• Inhibition of the CGRP pathway abolished beneficial effects of Mg implant on fracture healing, overexpression of CALCRL and RAMP1 enhanced them
					• Positive effect of CGRP on osteogenic differentiation of PDSCs in vitro enhanced by overexpression of *RAMP1* or *CALCRL* and inhibited by their knockdown
Hamushan et al. [49]	In vivo/in vitro	Femoral distraction osteogenesis model in Sprague-Dawley (SD) rats	Mg intramedullary pins	Stainless-steel nail	• Mg nail group: accelerated consolidation time; superior newly formed bone quality (significantly increased BMD, bone volume per total volume, bone mineralization); better biomechanical test results
					• RNA sequencing analysis: 430 differentially regulated genes in Mg group. Bioinformatic pathway analysis showed that Wnt pathway was the most significantly affected osteogenic-related pathway
					• Mg ions activate the alternative Wnt pathway through Hedgehog signaling
Go et al. [147]	In vivo	Bone defect in a rat humeral defect model	Mg(OH)_2_ in PLGA scaffolds	na	• Reduced inflammation
Diaz-Tocades et al. [50]	In vitro	na	na	na	• Addition of MgCl_2_ ions (magnesium chloride) dose-dependently increased protein expression of cyclin D1 and proliferating cell nuclear antigen
Liu et al. [51]	In vitro	na	na	na	• Incorporation of Mg into PEG-SH hydrogel increased cell viability and proliferation of BMSCs, as indicated by increased Ki67 protein expression and acridine orange staining
Hung et al. [52]	In vitro	na	na	na	• Significant increase in extracellular matrix mineralization and ALP expression in human BMSC cultures supplemented with 10 mM Mg compared to 0.8 mM Mg
					• Significant increase in the levels of active β-catenin (core intracellular mediator of Wnt signaling) in human BMSCs treated with Mg ions
					• Significantly up-regulated mRNA expression of *lymphoid enhancer-binding factor-1* (*LEF1*) and *dickkopf-related protein 1* (*DKK1*)
Cerqueira et al. [53]	In vitro	na	na	na	• Mg mainly regulates the expression of signaling pathways associated with osteogenesis, cell adhesion and oxidative stress; 21 pathways associated with osteogenesis were significantly affected by Mg, 16 pathways associated with cell adhesion
					• Addition of Mg to MT sol–gel improved adhesion of human osteoblasts to sol–gel coated titanium discs
Wang et al. [54]	In vitro	na	na	na	• Exposure of osteoblast-like MG-63 cells to 6 mM Mg ions resulted in increased proliferation and osteogenic differentiation
					• Involvement of the MAPK pathway in the promotion of osteogenesis induced by Mg ions
Ni et al. [55]	In vitro	na	na	na	• Mg exerts its osteogenic effect by regulating RUNX2 and SP7 through a p38-dependent mechanism
Lin et al. [65]	In vitro	na	na	na	• Enhancement of osteogenic differentiation in rat BMSCs by Mg ions, accompanied by an increase in the activation of MAPK1 and MAPK3
Shen et al. [66]	In vitro	na	na	na	• Mg ions have a positive effect on cell adhesion and migration on osteoblasts and/or their precursors
Yin et al. [67]	In vitro	na	na	na	• Inclusion of Mg in alginate hydrogels resulted in a significant increase in cell adhesion and stimulated morphological changes in adherent cells
Kim et al. [13]	In vitro	na	na	na	• Increased osteoblast migration into PCL/Mg scaffold
Zhang et al. [71]	In vitro	na	na	na	• Concentration of 4.1 mM significantly up-regulated the expression of VEGFA. This may indicate that Mg ions may, at least in part, promote angiogenesis in bone tissue by up-regulating VEGFA in macrophages
					• Concentration of 4.1 mM: switch in macrophage phenotype to M2, expression of osteogenic-related cytokines BMP2 and VEGF in macrophages

**Fig. 2.**
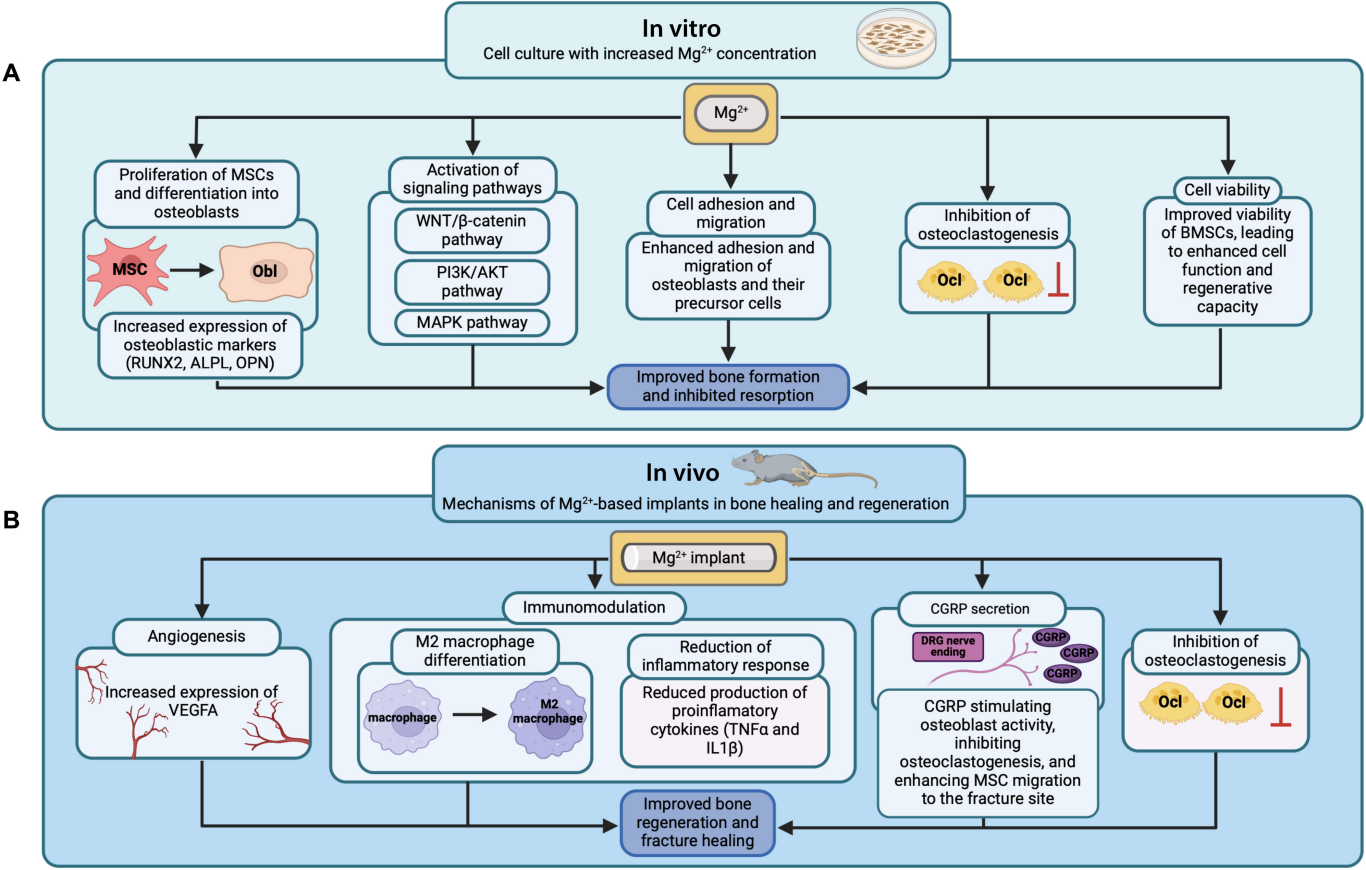
Mechanisms by which Mg-based implants promote bone healing and regeneration in in vitro and in vivo studies. (A) In vitro effects of Mg-based implants on bone cells. Mg (Mg^2+^) enhances the proliferation of mesenchymal stem cells (MSCs) and their differentiation into osteoblasts, leading to an increased expression of key osteoblastic markers (*RUNX2*, *ALPL*, and *OPN*). It also activates several key signaling pathways, including the WNT/β-catenin, PI3K/AKT, and MAPK pathways. Mg promotes cell adhesion and migration, supporting osteoblast function, while inhibiting osteoclastogenesis, which together improve bone formation and inhibit bone resorption. Moreover, Mg increases cell viability, enhancing the regenerative capacity of bone marrow stem cells (BMSCs). (B) In vivo mechanisms of Mg-based implants in bone healing and regeneration. Mg implants have been demonstrated to stimulate angiogenesis by increasing the expression of VEGFA and to modulate the immune response through the differentiation of macrophages into an anti-inflammatory M2 phenotype, thereby reducing the production of pro-inflammatory cytokines (TNFα and IL1β). Additionally, Mg has been shown to enhance CGRP secretion from dorsal root ganglia (DRG), which stimulates osteoblast activity, inhibits osteoclastogenesis, and enhances MSC migration to the fracture site. Overall, these combined biological effects of Mg are considered to result in improved bone regeneration and fracture healing. Artwork created with Biorender.com.

Han et al. [47] demonstrated that high-purity (HP) Mg screws (99.99 wt.% Mg) improved osseointegration compared to poly-l-lactic acid (PLLA) screws in a rabbit femoral intracondylar fracture fixation model. Significantly higher bone volume and bone mineral density (BMD) around the screws were observed in the HP-Mg group 8 weeks after surgery. In addition, the bone-to-implant contact area was significantly increased. Mg also accelerated bone formation in the fracture gap. In the HP-Mg group, woven bone was formed in the fracture gap at 4 weeks compared to 8 weeks in the PLLA group. Similarly, Hamushan et al. [49] reported that Mg intramedullary pins accelerated consolidation time in a femoral distraction osteogenesis model in Sprague–Dawley (SD) rats. Compared to the stainless-steel nail group and the negative control group, the Mg nail group showed superior newly formed bone quality as indicated by significantly increased BMD, bone volume per total volume, bone mineralization, and better biomechanical test results. Based on these observations, it is now well established that bioresorbable Mg ions derived from the insertion of Mg implants positively affects bone regeneration by inducing new bone formation. In the search for the underlying mechanisms, most mechanistic studies have focused on the impact of Mg ions derived from the insertion of Mg-based implants on osteogenesis, angiogenesis, and inflammation, as these 3 processes are essentially required for adequate bone healing (Fig. 2).

### Effects of Mg ions derived from the insertion of Mg-based implants on bone cells

Investigating the beneficial impact of Mg-based implants on bone formation, several researchers have reported Mg ions to positively affect the survival and proliferation of bone marrow stem cells (BMSCs) [47,50,51]. For instance, the addition of Mg ions (magnesium chloride) to cultures of rat BMSCs dose-dependently increased the protein expression of cyclin D1 and proliferating cell nuclear antigen, both well-known markers of cell proliferation [50]. Furthermore, incorporation of Mg into PEG-SH (polyethylenglycol compound containing sulfhydryl groups) hydrogel increased cell viability and proliferation of BMSCs, as indicated by increased Ki67 protein expression and acridine orange staining [51]. High concentrations of Mg ions (3.25 mM Mg, Mg screw extract) in human BMSC (hBMSC) cultures resulted in significantly increased cell viability compared to controls [47]. In addition, alkaline phosphatase (ALPL) activity and mRNA levels of osteoblastic markers *osteopontin* (*OPN*), *runt-related transcription factor 2* (*RUNX2*), and *ALPL* were significantly higher after 14 d in this study, suggesting enhanced osteoblastic differentiation [47]. Consistent with these findings, Hung et al. [52] demonstrated a significant increase in extracellular matrix mineralization (as indicated by alizarin red staining) and ALPL expression at 2 and 3 weeks, respectively, in hBMSC cultures supplemented with 10 mM Mg compared to 0.8 mM Mg. These observations are supported by several other studies that also reported the beneficial effects of Mg ions on osteoblastic differentiation in vitro [50,51,53–56].

**Fig. 3.**
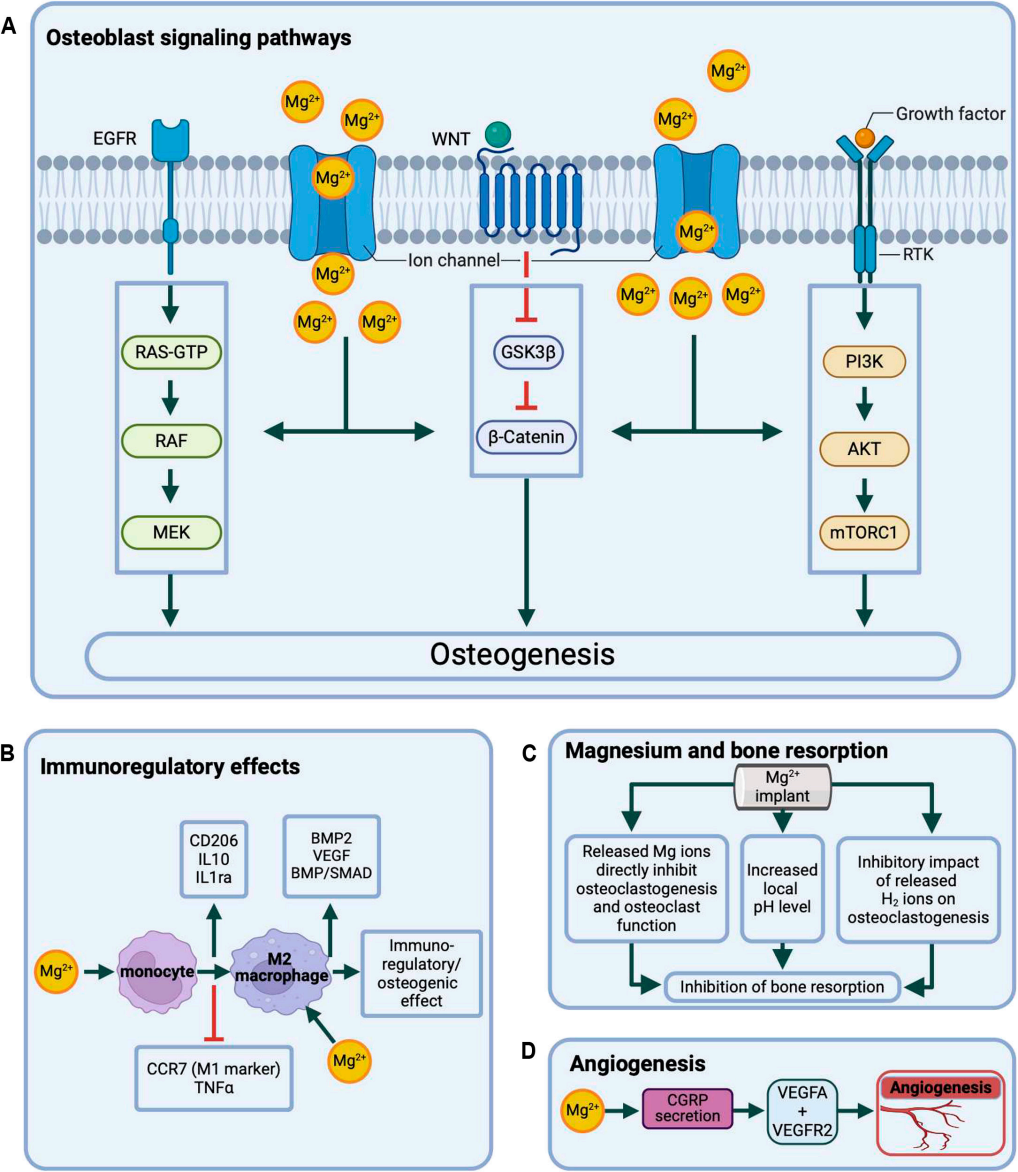
Influence of magnesium ions on osteoblasts, immune cells, and osteoclasts. (A) Magnesium ions (Mg^2+^) have been shown to exert pleiotropic effects on the osteogenic differentiation and migration of osteoblasts and their precursors. The 3 signaling pathways, including RAS/RAF/MEK/ERK, WNT/β-catenin, and PI3K/AKT/mTOR, are essential parts of a global cellular response induced by Mg exposure that is considered to ultimately enhance osteogenesis. (B) Mg induces a switch in macrophage phenotype to M2 and increase the expression of anti-inflammatory markers CD206, IL10, and IL1ra while decreasing the expression of pro-inflammatory CCR7 and TNFα. Mg ions promote the expression of osteogenic-related cytokines BMP2 and VEGF and activate the BMP/SMAD pathway. This results in an immunoregulatory and osteogenic effect. (C) Mg-based implants may inhibit bone resorption through (i) a direct inhibitory effect of Mg ions on osteoclastogenesis and osteoclast function, (ii) an increase in local pH levels, and (iii) an inhibitory impact of released H_2_ ions on osteoclastogenesis. (D) Mg ions enhance angiogenesis by stimulating the secretion of CGRP, which in turn up-regulates VEGFA and VEGFR2, key factors in the formation of new blood vessels. This angiogenic effect contributes to improved bone healing and regeneration.

From a mechanistic point of view, several pathways have been suggested to mediate the impact of Mg ions derived from the insertion of Mg-based implants on bone formation (Fig. 3A). In the previously mentioned study by Cerqueira et al. [53] on human osteoblasts, the Mg group showed differential expression of 144, 180, and 44 proteins after 1, 3, and 7 d, respectively. The PANTHER analysis showed that Mg regulated proteins in different functional classes and categorized the proteins into 19 classes, with the most dominant protein class being metabolite interconversion enzymes. Ingenuity pathway analysis (IPA) revealed that Mg mainly regulates the expression of signaling pathways associated with osteogenesis, cell adhesion, and oxidative stress. Specifically, 21 pathways associated with osteogenesis were significantly affected by Mg, including the mammalian target of rapamycin (mTOR), insulin-like growth factor 1 (IGF1), phosphatidylinositol 3-kinase (PI3K)/protein kinase B (AKT), RAS, mitogen-activated protein kinase (MAPK), and WNT pathways, while 16 pathways associated with cell adhesion were affected, with the p21-activated kinase (PAK) pathway being the most prominent. Cerqueira et al. [53] concluded that the effects of Mg on osteoblasts are rather complex, affecting the whole cellular machinery. Thus, the observed modulation of osteogenesis and cell adhesion by Mg was suggested to result from a global cellular response rather than from alterations in individual pathways.

On the contrary, Zhang et al. [14] proposed that the beneficial effect of Mg-based implants on osteogenesis is in most part mediated by the neuropeptide CGRP. This peptide, primarily expressed in sensory neurons innervating bone, has been shown to promote osteogenic differentiation of BMSCs and periosteum-derived stem cells (PDSCs) [14,57,58], to inhibit osteoclastogenesis [57,59], and to stimulate MSC migration to the fracture site [58]. Zhang et al. [14] found that inhibition of the CGRP pathway by injection of the CGRP receptor antagonist BIBN4096 and knockdown of the CGRP receptor components *calcitonin receptor-like receptor* (*CALCRL*) or *receptor activity modifying protein 1* (*RAMP1*) by small interfering RNA (siRNA) abolished the beneficial effects of Mg-based implant on fracture healing, whereas overexpression of *CALCRL* and *RAMP1* enhanced them. They also observed that the positive effect of CGRP on osteogenic differentiation of PDSCs in vitro was enhanced by overexpression of RAMP1 or CALCRL and inhibited by their knockdown [14]. Mechanistically, the authors observed that CGRP treatment of PDSCs increased the phosphorylation of cyclic adenosine monophosphate (cAMP) responsive element binding protein 1 (CREB1) and osterix (SP7), 2 important transcription factors during osteogenic differentiation of MSCs [60,61], which was again reversed by *CALCRL* knockdown [14]. Based on previous findings that CGRP activates the cAMP signaling pathway in rat brains and leads to CREB1 phosphorylation [62], Zhang et al. [14] concluded that CGRP induces its osteogenic effect through the cAMP signaling pathway and CREB1 phosphorylation.

Another study by Hung et al. [52] proposed that the osteogenic effect of Mg-based implants is exerted via the WNT/β-catenin pathway, which plays an essential role in regulating both bone formation and bone resorption in the skeleton. Immunocytochemical analysis showed an increased translocation of β-catenin to the nucleus in the presence of Mg ions, whereas in the control group β-catenin was predominantly located in the cytosol. Furthermore, gene expression analysis showed that Mg ions significantly up-regulated the mRNA expression of *lymphoid enhancer-binding factor-1* (*LEF1*) and *dickkopf-related protein 1* (*DKK1*), which are direct downstream target genes of active β-catenin [52]. These findings are supported by the study by Wang et al. [54], who found that exposure of osteoblast-like MG-63 cells to 6 mM Mg ions resulted in increased proliferation and osteogenic differentiation as evidenced by increased levels of osteogenic markers. When the protein expression levels of the Mg group were compared with the control, they found increased levels of inactive phosphorylated glycogen synthase kinase 3β (GSK3β) (a kinase that targets β-catenin for degradation) and β-catenin [54]. These findings suggest that the WNT/β-catenin signaling pathway was activated by Mg treatment. Finally, a further study supports the notion that Mg ions promote the stabilization and nuclear translocation of β-catenin in BMSCs through the phosphorylation of GSK3β [51].

Apart from the classical, i.e., canonical, WNT pathway, alternative WNT pathways and Hedgehog signaling have also been implicated in mediating the biological effects of Mg on osteogenesis. For example, in a rat distraction osteogenesis model, Hamushan et al. [49] compared gene expression between a Mg pin group, a stainless-steel pin group, and a negative control group after 6 weeks of implantation. RNA sequencing analysis revealed 430 differentially regulated genes in the Mg group. Bioinformatic pathway analysis [Kyoto Encyclopedia of Genes and Genomes (KEGG)] showed that the WNT pathway was the most significantly affected osteogenic-related pathway. However, quantitative reverse transcription polymerase chain reaction (qRT-PCR) revealed no significant difference in the mRNA expression of the canonical WNT pathway genes *WNT3A* and *β-catenin*, while the mRNA expression of the alternative WNT pathway genes such as *WNT5B*, *frizzled receptor* (*FZD*), *yes-associated protein* (*YAP*), and *RUNX2* was increased in the Mg group, indicating the activation of the alternative WNT pathway. Western blot and immunohistochemical analysis supported these findings. Further analysis using GCBI (gene-cloud of biotechnology information) Gene Radar identified 25 upstream genes of *WNT5B*, of which only 2 genes, *GLI family zinc finger 1* (*GLI1*) and *GLI family zinc finger 2* (*GLI2*), were differentially expressed in RNA-sequencing analysis. These 2 genes are downstream target transcription factors of the Hedgehog signaling pathway. qRT-PCR confirmed the increased expression of *GLI1* and *GLI2*, as well as the increased mRNA expression of *PTCH1* and *Smoothened*, with PTCH1 encoding a receptor protein that releases its inhibitory effect on Smoothened protein upon binding of Hedgehog pathway ligand. The mRNA expression of sonic Hedgehog and Indian hedgehog ligands remained unchanged. Western blot and immunohistochemical analysis supported the qRT-PCR results. Thus, Hamushan et al. [49] concluded that Mg ions activate the alternative WNT pathway through Hedgehog signaling, and in this regard, the authors also confirmed that Mg ions can bind to the PTCH1 protein using molecular dynamics simulations.

Based on its pivotal importance in many key cellular processes, several studies have also investigated the involvement of the PI3K/AKT signaling pathway in mediating the positive effects of Mg ions on the osteogenic differentiation of stem cells [51,53,63]. Liu et al. [51] showed that inhibition of PI3K using LY294002 reversed the increased expression of β-catenin, phosphorylated AKT (pAKT), and pGSK3β induced by Mg ions in BMSCs. Another study found that inhibition of PI3K with LY294002 reversed the beneficial effects of Mg ions on motility and osteogenic differentiation in human osteoblasts (hFOB1.19) [63]. Mg-induced increases in wound scratch assay and ALPL staining, as well as up-regulation of gene expression levels of the migration-related genes *matrix metallopeptidase 2* (*MMP2*), *MMP9*, and *vascular endothelial growth factor A* (*VEGFA*) and the osteogenic-related genes *RUNX2* and *ALPL*, were all reversed by PI3K inhibition [63]. However, it should be reiterated that the use of LY294002 as a tool to study the role of the PI3K pathway in cellular processes is not optimal as it is a nonselective inhibitor of PI3K [64]. To the best of our knowledge, no study has employed a specific PI3K inhibitor to investigate its involvement in the osteogenic effect of Mg ions to date.

Apart from PI3K/AKT signaling pathway, several studies also provide evidence supporting the involvement of the MAPK pathway in the promotion of osteogenesis induced by Mg ions [53,55,56,65]. According to the research by Wang et al. [56], the enhancement of osteogenic differentiation in hBMSCs by Mg ions was accompanied by a significant increase in the mRNA expression of *extracellular signal-regulated kinase 2* (*ERK2*), *c-Jun N-terminal kinase 1* (*JNK1*), *JNK2*, and *p38*, which are all kinases of the MAPK family. Western blot analysis also showed increased activation (i.e., phosphorylation) of these proteins. The positive effect of Mg ions on the osteogenic differentiation of hBMSCs was reversed by inhibition of MAPK1, p38, and JNK with FR180204, SB203580, and SP600125, respectively, with p38 inhibition having the most significant negative effect. Furthermore, gene silencing of p38 also reduced the beneficial osteogenic effect of Mg ions [56]. Ni et al. [55] observed that the positive effect of Mg ions on osteogenic differentiation in mouse MSCs, as indicated by increased protein expression of RUNX2 and SP7, was accompanied by an increase in activated p38 protein expression. In addition, they found that Mg ions increased p38 activation in a time-dependent manner. Gene expression analysis revealed that Mg ions also increased the mRNA expression of p38 downstream target genes including *heat shock protein 27* (*HSP27*), *activating transcription factor 4* (*ATF4*), *DNA damage inducible transcript 3* (*DDIT3*), and *myocyte-specific enhancer factor 2C* (*MEF2C*). In turn, inhibition of p38 with SB203580 blocked the Mg-induced enhancement of osteogenic differentiation and up-regulation of p38 target genes. Therefore, Ni et al. [55] concluded that Mg exerts its osteogenic effect by regulating *RUNX2* and *SP7* through a p38-dependent mechanism. Finally, Lin et al. [65] reported that the enhancement of osteogenic differentiation in rat BMSCs by Mg ions, as indicated by increased ALPL activity and osteocalcin (OCN) protein expression, was accompanied by an increase in the activation of MAPK1 and MAPK3 (also known as ERK1), while other MAPK family proteins remained unaffected. To confirm the role of MAPK1/3 in Mg-induced osteogenic differentiation, a selective inhibitor of MAPK1/3 (PD98059) was added to the cultures and was found to reverse the Mg-induced increase in osteogenic differentiation [65].

In addition to regulating osteogenesis directly, Mg ions have been found to have a positive effect on cell adhesion and migration on osteoblasts and/or their precursors [66]. In a study using mouse MC3T3-E1 pre-osteoblasts, it was found that the presence of Mg ions at concentrations of 4.11, 8.22, and 16.44 mM significantly increased cell adhesion compared to controls [66]. Cells also showed a change in morphology, forming more processes at a faster rate. In addition, Mg ions significantly stimulated migration in a cell migration assay. The group at 4.11 mM Mg ion concentration had the highest mean cell velocity, with further increases in Mg ion concentration decreasing the mean cell velocity [66]. Consistent with these findings, Yin et al. [67] conducted a study on the same cell line and found that the inclusion of Mg in alginate hydrogels resulted in a significant increase in cell adhesion and stimulated morphological changes in adherent cells. Similarly, Cerqueira et al. [53] found that the addition of 1.5 wt % Mg to MT sol–gel (a mixture of methyltrimethoxysilane and tetraethyl orthosilicate) improved the adhesion of human osteoblasts to sol–gel coated titanium discs. Confocal fluorescence imaging after 7 d of differentiation showed a larger area of cells in contact with the disc coating in the Mg group compared to the control. Osteoblasts in the Mg group had a more elongated shape and more protruding lamellipodia. Gene expression analysis showed a significant increase in the expression of the adhesion-related genes *matrix remodeling associated 8* (*MXRA8*) and *fibrillin-1* (*FBN1*) [53]. Kim et al. [13] investigated the impact of Mg incorporation into polycaprolactone (PCL) scaffolds (PCL:MgCl_2_ 9:1 wt %) on osteoblast migration within the scaffold. They observed increased osteoblast migration into the PCL/Mg scaffold, which was supported by immunofluorescent tubulin staining and an increase in the amount of DNA and protein extracted from the inside of the scaffolds.

Taken together, the various results of the above studies suggest that Mg ions derived from the insertion of Mg-based implants exert pleiotropic functions on the osteogenic differentiation and migration of osteoblasts and their precursors. However, further in vivo and in vitro studies are required to delineate the precise roles of the suggested cellular mechanisms and molecular pathways in more detail.

### Effects of Mg ions derived from the insertion of Mg-based implants on soft tissue

Recent studies have discovered a vessel subtype, type H vessels, that couples angiogenesis and osteogenesis [68]. A decrease in type H vessels is associated with loss of bone mass and low bone formation [69], and insufficient type H vessel formation in the fracture callus results in impaired bone regeneration. Therefore, this neovascularization–osteoblast regulatory mechanism is also of key importance for biodegradable orthopedic implants. As Mg-based implants were found to stimulate secretion of the vasoactive peptide CGRP in bone tissue [14,48], several studies have assessed the impact of Mg on angiogenesis in vivo and in vitro (Table 2).

Ye et al. [48] conducted a study on the effect of complementary intramedullary Mg nail implantation on distraction osteogenesis in a rat model of a critical size mid-shaft femoral defect. The Mg group showed higher CGRP expression in the periosteum and increased angiogenesis in vivo. The authors also investigated the effect of CGRP on angiogenesis in vitro. Scratch tests and tube formation assays showed a CGRP dose-dependent increase in the migration and tube formation of mouse endothelial cells. In addition, gene expression of *VEGFA* and *VEGF receptor type 2* (*VEGFR2*) was increased, suggesting that CGRP stimulates angiogenesis in vitro. They concluded that Mg increases angiogenesis in vivo through CGRP signaling [48]. Consistent with the findings of Ye et al. [48], Zhang et al. [14] reported that intramedullary Mg rods resulted in increased CGRP levels in peripheral cortical bone and increased CGRP expression in L4 dorsal root ganglia (DRGs). In addition, the presence of Mg ions in DRG neuron cultures increased vesicle aggregation in neuronal terminals and intracellular adenosine triphosphate (ATP) concentration [14]. Cytochalasin B, an inhibitor of actin polymerization, reversed the increased vesicle aggregation, which, together with the knowledge that CGRP is transported in vesicles from the neuronal body of DRGs to the synaptic terminal [70], suggests that Mg ions may enhance ATP-facilitated actin polymerization, thereby promoting the transport of CGRP-containing vesicles to synaptic terminals.

In a separate study, Zhang et al. [71] investigated the effect of different concentrations of Mg ions on RAW264.7, a murine macrophage cell line, and found that a concentration of 4.1 mM significantly up-regulated the expression of *VEGFA*. Since *VEGFA* plays a critical role in promoting neovascularization in bone tissue, the authors concluded that Mg ions may, at least in part, promote angiogenesis in bone tissue by up-regulating *VEGFA* in macrophages [72]. Mi et al. [73] conducted a study using a rat distraction osteogenesis model where they injected exogenous CGRP directly into the bone defect after each distraction cycle. The results showed that CGRP increased the population of endothelial progenitor cells (EPCs) in the bone defect region and stimulated blood vessel formation. Vessel volume and vessel volume per tissue volume were significantly greater in the CGRP group than in the control group. In addition, in an in vitro test, administration of 10 nM CGRP significantly increased the mRNA levels of *CD31* and *CD144* during endothelial differentiation of BMSCs, indicating increased formation of EPCs [73]. To evaluate the effect of EPCs on the osteogenic differentiation of BMSCs, direct coculture of EPCs with BMSCs cultured in a 1:1 mixture of osteogenic and angiogenic media was performed. The results showed that cocultures with EPCs exhibited a larger area of ALPL staining, indicating enhanced osteogenic differentiation. qRT-PCR results showed that cocultures with EPCs had significantly higher gene expression of *ALPL*, *BMP2*, and *RUNX2* compared to BMSC monocultures, suggesting that CGRP has an indirect positive effect on osteoblastic differentiation through its angiogenic effect [73]. Several other studies have found that Mg ions enhance the expression of *VEGF*, *MMP2*, and *MMP9*, all of which are involved in neoangiogenesis, in both osteoblasts and hBMSCs [63,65,74]. Mg ions have also been found to significantly improve the ability of BMSCs to attract endothelial cells, as observed in a trans-well migration assay [65]. Furthermore, implantation of Mg-enriched spheroids into rat cranial defects was found to enhance vascularization in vivo [65].

From a mechanistic point of view, an involvement of the CGRP–focal adhesion kinase (FAK)–VEGFA signaling axis in the angiogenic effect of Mg-based implants has been suggested [48]. As mentioned above, Ye et al. [48] showed in their in vitro experiments that CGRP promoted tube formation and enhanced the migration of mouse endothelial cells. This effect was accompanied by the up-regulation of *VEGFA* and *VEGFR2* and an increase in FAK phosphorylation. The role of the CGRP-FAK-VEGF signaling axis was verified in a subsequent in vivo experiment. Here, the beneficial effect of the Mg-based implant on angiogenesis in vivo was reversed by inhibition of CGRP, FAK, and VEGFR2 using the CGRP receptor antagonist BIBN4096, Y15 (FAK inhibitor), and SU5416 (VEGFR2 inhibitor), respectively [48]. These treatment approaches also reversed the beneficial effect of the Mg-based implant on bone formation in the defect gap in vivo. In contrast, Mi et al. [73] proposed that the aforementioned beneficial effect of CGRP on the differentiation of BMSCs into EPCs is mediated by the PI3K-AKT signaling pathway, as the protein expression of pAKT/AKT and pPI3K was significantly up-regulated in the presence of CGRP. Inhibition of PI3K with LY294002 reversed the increased epithelial differentiation, and the expression levels of pAKT/AKT and pPI3K were significantly suppressed compared to the CGRP without inhibitor group [73]. However, while the increased expression of pAKT/AKT and pPI3K may suggest that CGRP activates the PI3K/AKT pathway, the use of LY294002, a nonselective inhibitor of PI3K, is insufficient to confirm this [64].

Therefore, collective evidence suggests that Mg-based implants stimulate bone formation not only by directly promoting osteogenesis of MSCs but also by the promotion of angiogenesis in the skeleton, in particular during bone regeneration. However, the extent to which enhanced angiogenesis accounts for the beneficial impact of Mg ions derived from the insertion of Mg-based implants on bone formation and the precise cellular and molecular mechanisms underlying this effect remain to be elucidated.

### Effects of Mg ions derived from the insertion of Mg-based implants on blood cells

Bone remodeling and bone regeneration are regulated not only by classic bone cells including osteoblasts and osteoclasts but also by immune cells. This is best illustrated clinically, as many patients with immune disorders display bone pathologies including increased fracture risk and elevated incidence of fracture nonunion. Based on the essential function of Mg for optimal immune function and regulation of inflammation, several studies assessed the impact of Mg on immune cells (Fig. 3B). In both bone remodeling and regeneration, macrophages play a critical role and specifically M2 macrophages (anti-inflammatory) have been shown to enhance osteoblastic differentiation of MSCs, thereby promoting osteogenesis [75,76]. In the study by Zhang et al. [71], the authors investigated the effect of Mg ion concentration on murine macrophage cell lines. They found that 4.1 mM Mg was the most effective in terms of producing an anti-inflammatory and osteogenic effect and inducing a switch in macrophage phenotype to M2. At this concentration, Mg ions increased the expression of CD206 (a M2 marker) and the anti-inflammatory cytokines IL10 and IL1ra (IL1 receptor antagonist) while decreasing the expression of C–C motif chemokine receptor 7 (CCR7) (an M1 marker) and the pro-inflammatory cytokine TNFα [71]. The effect of Mg ions on macrophages was mediated through toll-like receptor (TLR) and nuclear factor κB (NFκB) signaling pathways, as evidenced by the significant down-regulation of TLR adapter proteins [myeloid differentiation primary response 88 (MYD88), toll-like receptor adaptor molecule 1 (TICAM1), and TICAM2]. This led to the activation of NFκB and a significant up-regulation of IκBα (nuclear factor of κ light polypeptide gene enhancer in B cells inhibitor α), a downstream protein that binds NFκB and inhibits its nuclear translocation [71]. In addition, Mg ions promoted the expression of the osteogenic-related cytokines BMP2 and VEGF in macrophages. To confirm this effect, BMSCs were cultured in conditioned medium obtained from macrophage cells exposed to high concentrations of Mg ions, which significantly increased ALPL activity and gene expression of *RUNX2*, *ALPL*, *OPN*, and *OCN*. The increased gene and protein expression of SMAD4, SMAD5, and BMPR1A also suggested that the osteogenic effect on BMSCs mediated by macrophages in response to Mg ions was achieved through activation of the BMP/SMAD pathway [71]. The immunomodulatory and pro-angiogenic effect of Mg have also been demonstrated by Amara et al. [77] in a rat soft tissue model in vivo. In their study, the initial Mg-based implant degradation amplified inflammation, but not cytotoxicity, and promoted vascularization. Positive associations were shown between Mg and the expression of inducible nitric oxide synthase (iNOS) and TLR4. On the contrary, a negative association was observed between Mg and fibroblast growth factor 2 (FGF2) expression.

Thus, some evidence is available that Mg-based implants may exert immunomodulatory effects in the bone microenvironment, with potential beneficial effect on bone regeneration, and further studies are warranted to delineate the precise impact of Mg on inflammatory processes relevant for bone biology. Here, it will be interesting to also study other immune cell populations, including neutrophils and mast cells, which are known to profoundly affect regenerative processes in the skeleton.

## Effects of Mg-Based Implant Degradation Products

As discussed below, the occurrence and nature of radiolucent zones around Mg-based implants, which contradicts the positive effects of released Mg ions derived from the insertion of Mg-based implants on osteogenesis as summarized before, is still not fully understood and delays more widespread clinical use of bioresorbable Mg ions derived from the insertion of Mg-based implants due to potential safety concerns. As radiolucent zones may radiologically present similar to classic osteolysis, usually caused by focally excessive bone resorption, the question arouse whether Mg itself or its degradation products may directly or indirectly stimulate osteoclast formation and function. For that, an influence of the 3 main degradation products of Mg-based implants, namely, Mg ions, H_2_ gas, and a pH increase associated with the formation of Mg hydroxide molecules, have been studied for their potential to influence osteoclastogenesis and osteoclast activity (Fig. 3C) [78]. The release of solid particles during Mg degradation in vivo is even described to attract macrophages that may negatively impact osseointegration [79].

To date, numerous in vitro studies have investigated the effect of Mg ions on various osteoclastic cell culture models, with conflicting results and significant variability in osteoclastic differentiation and activity [80–87]. This variability can be attributed to factors such as the source of Mg ions, stage of cell differentiation, culture models, concentration ranges of Mg ions, and pH of the culture medium, among other potential variables [80]. One study found that the differentiation of RAW264.7 macrophages into multinucleated tartrate-resistant acid phosphatase (TRAP)-positive osteoclasts was significantly inhibited at a concentration of 10 mM Mg ions [81]. In addition, high concentrations of Mg ions also inhibited the activity of mature osteoclasts cultured on dentin slices, as evidenced by a reduction in the number of resorption pits formed, while the number of osteoclast cells remained unchanged [81]. Of note, this study used pure Mg ingots as the source of Mg ions and did not control for pH levels, which are bound to increase due to pure Mg degradation [78]. Increased alkalinity is known to inhibit osteoclast activity, thus potentially contradicting the observed effects [88,89]. Another study found a significant increase in the number of TRAP-positive multinucleated osteoclasts differentiated from bone marrow cells cultured on plastic and bone slices when the concentration of Mg ions in the culture medium was reduced to 0.4, 0.08, and 0 mM compared to the basal Mg ion concentration of 0.8 mM [82]. Mg deficiency was found to increase the expression of osteoclastogenic-related genes in bone marrow cells cultured on plastic but not on bone. Interestingly, Mg deficiency reduced the resorption and TRAP activity of single multinucleated osteoclasts, while the overall resorption activity remained unchanged due to a higher number of osteoclast cells [82]. Similarly, Zhai et al. [83] found that Mg leach liquor (MLL), obtained by incubating pure Mg in culture medium, significantly reduced the number of TRAP-positive multinucleated osteoclasts differentiated from bone marrow macrophages (BMMs). When the alkalinity of the MLL was controlled for, either by including a NaOH control group or by neutralizing the pH of the MLL to the physiological level of 7.4, high concentrations of Mg ions continued to significantly inhibit osteoclastogenesis in a dose-dependent manner [83]. Western blot and immunofluorescence analysis showed that MLL inhibited RANKL-induced activation and nuclear translocation of NFκB and up-regulation of NFATc1 (nuclear factor of activated T cells 1) expression, a master regulator of osteoclastogenesis. Mechanistically, MLL inhibition of osteoclast formation was mediated by preventing RANKL-induced phosphorylation and degradation of the inhibitory subunit of NFκB, IκBα [83].

Differing from the results described above, a study comparing different sources of Mg ions found that the addition of MgCl_2_ to peripheral blood mononuclear cell (PBMC) cultures resulted in a significant increase in the number of differentiated TRAP-positive osteoclasts up to a concentration of 15 mM [84]. However, further increasing the concentration to 25 mM resulted in a significant decrease in the number of differentiated osteoclasts. Also, pure Mg extract was found to significantly reduce the number of TRAP-positive osteoclasts. In a bone resorption assay, where total resorption activity was normalized to osteoclast numbers, MgCl_2_ had no significant effect on bone resorption up to a concentration of 15 mM, whereas a concentration of 25 mM significantly inhibited bone resorption. However, pure Mg extract significantly increased the average resorption activity of individual osteoclasts, although the study did not provide information on total resorptive activity [84]. In another in vitro study investigating the impact of pure Mg extract on cocultures of osteoblasts and PBMCs in the absence of exogenous osteoclastogenic inducers [recombinant macrophage colony-stimulating factor (M-CSF) and RANKL], it was found that osteoclastogenesis was slightly enhanced up to a concentration of 6 mM Mg, as evidenced by the TRAP activity assay and the expression of osteoclastogenic marker genes [85]. However, higher concentrations of Mg ions resulted in a significant inhibition of osteoclastogenesis.

In contrast to the studies mentioned above, Mammoli et al. [86] did not use exogenous M-CSF and RANKL to induce osteoclastogenic differentiation of progenitor cells when investigating the effect of Mg on osteoclastogenesis. Instead, they treated osteoclastic progenitor cell line (U937 cell line) with phorbol 12-myristate 13-acetate (PMA) and 1,25(OH)_2_ vitamin D_3_. They found that high concentrations of Mg ions significantly enhanced PMA/1,25(OH)_2_ vitamin D_3_-induced osteoclastogenic differentiation, as indicated by increased expression levels of osteoclastogenic-related genes (transcription factors and differentiation markers) and the number of osteoclasts measured by flow cytometry [86]. However, the study did not specify the source of Mg ions or report pH values in respective cell cultures.

Another possible way of Mg-based implants to influence osteoclastogenesis is the increase in pH associated with the formation of Mg(OH)_2_ [78]. Zhai et al. [83] observed that, regardless of the alkaline agent used (pure Mg or NaOH), a higher pH was associated with a decrease in the number of osteoclasts differentiated from BMMs. Two separate studies reported that increasing the pH of the culture medium beyond the physiological value of 7.4 significantly suppressed osteoclastogenic differentiation and function of RANKL-stimulated RAW264.7 macrophages [88,89]. These effects were assessed by measuring the surface area of TRAP-positive multinucleated cells, expression levels of osteoclastogenic-related genes, and resorption pit area. However, it remains unclear to what extend Mg-based implants also affect local pH levels in vivo, and studies with in situ pH measurements are required to further delineate potential biological implications.

In addition to alkalinity and Mg ions, hydrogen (H_2_) gas has been suggested to influence osteoclast function [90–92]. Liu et al. [90] found that high levels of H_2_ gas in the environment of bone marrow mononuclear cells cultured with osteoclastogenic inducers (M-CSF and RANKL) significantly inhibited osteoclastogenesis and bone resorption activity. However, this inhibitory effect was only observed at extremely high H_2_ gas concentrations (25%, 50%, and 75%), which are unlikely to be physiologically present, as hydrogen gas has been shown to be rapidly exchanged and present at low concentrations in peri-implant gas cavities shortly after implantation [93]. A similar study found that H_2_ gas was not cytotoxic to RAW264.7 macrophages at concentrations up to 80% and that H_2_ gas at 60% inhibited osteoclastogenesis and bone resorption activity of RANKL-stimulated macrophages in vitro [91]. Further analysis by immunoblotting and immunofluorescence showed that H_2_ inhibited RANKL-induced activation and nuclear translocation of NFκB.

While in vitro studies have shown that degradation products of Mg-based implants can inhibit osteoclastic activity, it is unlikely that this effect will be observed to the same extent in vivo. Here, bone and immune cells are subject to complex intercellular communication, and osteoclastogenesis is tightly coupled to osteogenesis [9]. Osteoclasts play a critical role in bone remodeling and fracture healing, even in the early stages, and impairment of osteoclastic function has been shown to delay fracture healing [94–96]. In vivo studies investigating the effect of Mg-based implants on osteoclastogenesis and bone resorption activity have yielded conflicting results, much like in vitro studies. While some studies have found no significant effect of Mg on osteoclast activity, others have reported either a decrease or an increase in activity [87,97–100].

Jahn et al. [87] compared Mg alloy (Mg_2_Ag; 2 wt % Ag) and steel intramedullary nails in a mouse mid-shaft femoral fracture model. They found that at 21 d after surgery, there was a significant decrease in both bone resorption activity, measured as eroded surface per bone surface, and osteoclast cell number in the bone tissue surrounding the implant in the Mg alloy group. The reduced bone resorption activity and increased osteoblastic activity in the Mg alloy group resulted in increased callus formation [87]. In a study using a rat model of ovariectomy-induced osteoporosis, a femoral osteotomy was performed and fixed with either stainless-steel or Mg alloy pins (JDBM alloy coated with brushite and referred to as Mg/CaP) [98]. Analysis of femoral samples 12 weeks after surgery using TRAP immunoblotting showed no significant difference in the number of osteoclast cells between the Mg alloy and stainless-steel pin groups [98]. Another study comparing Mg alloy and PLLA pins implanted in pre-drilled holes in rat tibiae reported no statistically significant change in TRAP-positive osteoclast surface area at 6 weeks after surgery [99]. Witte et al. [97] conducted a study comparing Mg alloy scaffolds and autologous bone grafts implanted in rabbit femoral condyle defects. They observed that the Mg alloy group had a significantly greater bone surface area occupied by TRAP-positive osteoclasts at both 3 and 6 months after surgery, indicating increased osteoclastic activity. However, it is noteworthy that the Mg alloy group also had higher osteoblastic activity, bone volume per tissue volume, and a more mature bone structure as evidenced by a higher trabecular number and lower trabecular separation. Therefore, the increased osteoclastic activity around the Mg alloy seems to promote bone maturation rather than hinder fracture healing [97].

Thormann et al. [100] conducted a study comparing the effects of Mg alloy, titanium, and PLLA screws implanted in pre-drilled holes in the epiphysis of sheep distal femura. They reported a much higher number of TRAP-positive multinucleated osteoclasts and macrophages in the Mg alloy group, mainly located around the gas cavities. This suggests that the excessive osteoclastogenesis at these sites may result from inflammatory responses triggered by gas expansion, rather than a physiological response in bone remodeling. However, the study did not comment on the significance of the difference in osteoclast numbers and did not provide further data in this regard [100]. Two other noteworthy studies, although not directly investigating the effect of Mg-based implants on bone resorption, have investigated the effect of degradation products of Mg-based implants on osteoclasts in vivo [83,101]. First, Janning et al. [101] found that implantation of cylinders of pure Mg(OH)_2_ powder, prepared using a cold isostatic pressure method, into a defect site in the rabbit femoral condyle significantly reduced the number of osteoclasts at the defect site compared with the negative control group, in which the defect was left empty, at 2 and 4 weeks after surgery. By 6 weeks after surgery, the number of osteoclasts had returned to normal levels [101]. Second, another study found Mg leached liquor to have a significant suppressive effect on bone resorption caused by titanium wear particle osteolysis in a mouse calvarial model [83]. Here, Mg leached liquor, injected into the calvarial periosteum every other day, significantly reduced the number of osteoclasts at the site of titanium particle implantation, as later shown by histochemical analysis.

In summary, most available literature suggests a moderate inhibitory effect of Mg ions derived from the insertion of Mg-based implants and its degradation products on osteoclastogenesis and bone resorption. While this may affect remodeling of regenerating bone, it is unlikely that excessive bone resorption, or even osteolysis, induced by Mg-based implants is causative for the occurrence of the clinically observed radiolucent zones, as discussed below.

## Challenges, Recent Advances, and Perspectives

Despite increasing research and a rising clinical acceptance of Mg-based implants, there are still several challenges for commercialization, especially in regard to weight-bearing skeletal sites. Mg-based implants have demonstrated several and significant beneficial effects on fracture healing, including direct promotion of osteogenesis and indirect promotion through stimulation of angiogenesis, cell motility, and immunomodulation. The signaling mechanisms responsible for these beneficial effects are complex, and most likely involve multiple signaling pathways and intercellular communications. Recent studies clarified numerous signaling mechanisms; however, further research is urgently needed to fully comprehend the biological effects of Mg-based implants.

Another important aspect in the future clinical use of Mg-based implants is the possibility of additive manufacturing. Similar to other metals or metal alloys, it has now become possible to 3-dimensionally (3D) print individualized Mg-based implants for various surgical indications. Employed techniques include powder bed fusion, wire arc AM, paste extrusion deposition, friction stir AM, and jetting technologies (reviewed in detail by Karunakaran et al. [102]). However, Mg alloys are difficult to 3D print due to the high chemical reactivity, which may pose a significant risk of combustion. Therefore, to ensure safe material handling, specialized equipment is required to print Mg in inert atmosphere. Nevertheless, 3D printing of Mg-based implants and subsequent modification of surface coatings will allow for more intricate geometries and novel designs that may improve implant performance in the long term.

Most importantly, the formation of peri-implant radiolucent zones, being part of the physiological biodegradation process and apparently not negatively affecting clinical outcomes, requires further clarification. Formation of temporary radiolucent zones around Mg-based implants seems to be promoted by rapid degradation of the Mg alloy. In the presence of water, Mg degrades through a corrosion reaction, which produces Mg hydroxide and H_2_ gas, as shown in the equation Mg + 2H_2_O ➔ Mg(OH)_2_ + H_2_ [78,103,104]. In case the degradation rate of a Mg-based implant exceeds the body's ability to eliminate the degradation products, hydrogen gas can accumulate within the tissue and form gas cavities. This phenomenon is currently believed to be the reason for appearance of radiolucent zones around the implants that are visible in radiographs [103,105–107]. Assessing the performance of Mg-based implants in our own department, we monitored postsurgical outcomes of patients with elbow injuries. Here, the temporary occurrence of radiolucency became quite prominent in sizes, as demonstrated exemplary by serial cone beam CT imaging of a patient with a distal humerus fracture treated with Mg-based screws (Fig. S1). Although we do not have long-term follow-up data on this particular patient, the occurrence of radiolucent zones appears to be of temporary nature and not to affect bone healing. Nevertheless, the occurrence of radiolucent zones requires further study whether gas formation can lead to clinical complications such as implant loosening or interfere with osteoconduction by impeding the implant's contact with the surrounding tissue. Further, it is also possible that the physical pressure and/or content of these gas cavities may induce some type of inflammatory response that promotes pathologic bone resorption and inadequate bone formation. Thormann et al. [100] described in their in vivo study cavities surrounded by fibrous tissue in the peri-implant space after the degradation of Mg-based screws. The formation of the cavities was accompanied by inflammatory responses with a high number of macrophages, the precursors of osteoclasts. Thus, this may have attributed to cavity formation by a combination of the released gas and the detached intermediate degradation products of the Mg-based implants, which can attract macrophages [100]. A faster degradation rate of the Mg-based implants led to more accumulation of both gas and detached particles. Negligible cavities were observed in coated Mg-based implants [100]. In this respect, Kraus et al. [104] compared the effects of rapidly and slowly degrading Mg alloys implanted in rat femura. The rapidly degrading alloy resulted in significant hydrogen evolution and gas cavitation, which impeded bone healing and increased formation of fibrous tissue and callus. Complete consolidation of the cortical bone defect was achieved only after complete implant degradation and gas resorption. In contrast, the slowly degrading alloy caused minimal gas evolution and did not interfere with tissue healing. In addition, the slowly degrading implant showed better osteoconductivity and formed a tight connection with the surrounding bone tissue, resulting in better bone regeneration [104].

These findings highlight the significance of implant degradation rate in determining the success of bone tissue regeneration. The current scientific consensus is that the degradation rate of Mg-based implants must be optimized so that they maintain their mechanical integrity in the initial phase, allowing them to provide sufficient stabilization of the fracture site until the bone has consolidated sufficiently to bear the load. In addition, it is important that Mg-based implants do not degrade at a rate that interferes with bone healing by generating excessive amounts of gas and/or other degradation products that adversely interfere with tissue regeneration. Producing Mg-based implants with appropriate degradation characteristics is currently achieved by using alloying and surface modification techniques [12]. The MAGNEZIX implant (Syntellix), which consists of a Mg alloy containing Mg, zirconium, and rare earth metals (MgYREZr), is one option for Mg-based implants and appears to have an adequate degradation rate for clinical use. MAGNEZIX is already used in clinical practice, and several clinical studies with MAGNEZIX implants have reported the formation of peri-implant radiolucent zones that did not affect the clinical outcome or led to complications, and gradually resolved on their own [15,108–117]. However, 2 articles describe excessive degradation of MAGNEZIX implants. A case report by Wichelhaus et al. [118] reports the failure of MAGNEZIX implants used in a scaphotrapezotrapezoidal (STT) arthrodesis procedure. The complex background of the patient, including scaphoid fracture and STT osteoarthritis, suggested though that the immune reactions occurring in the STT joint may have influenced the inappropriately rapid degradation and loss of mechanical integrity of the Mg-based implant [118]. In another clinical study, MAGNEZIX implants were used to fix acute scaphoid fractures [119]. Despite excellent functional and clinical outcome with no complications, the study was stopped because of extensive radiolucent zone formation in 3 of the 5 patients, which the authors interpreted as “osteolytic resorption cysts” [119]. According to Holweg et al. [113], small bones have a higher risk of implant failure due to the accumulation of trapped gas. This is because they have a reduced capacity to disperse the gas that develops. However, even in the small scaphoid bone, recent clinical studies have shown excellent functional and clinical results for Mg-based implants [116,117]. Both studies report the transient formation of peri-implant radiolucent zones, which had no clinical significance and did not affect outcomes.

Another CE-certified (Conformité Européenne) and clinically applied Mg-based implant is based on the mm.X implant technology by medical magnesium (Medical Magnesium GmbH, Aachen, Germany) (Fig. S2). Here, the Mg alloy WE43MEO (Meotec GmbH, Aachen, Germany) with a generally improved corrosion resistance is used, which can be attributed to its rare earth and zirconium content and strict impurity control. Additionally, the surface of mm.X implants is modified by means of a plasma electrolytic oxidization process (KERMASORB), where an oxidized surface layer is generated on the alloy to further retard the corrosion [120]. mm.X implants have been showing promising results in in vitro studies. In a biomechanical testing of the mm.PIP versus K-wire for the surgical correction of hammertoe deformities with arthrodesis of the proximal interphalangeal joint, the biomechanical properties of the mm.PIP were found to be comparable to the biomechanical properties of a K-wire [121]. First clinical data are reported in a case report of the treatment of an osteochondrosis dissecans in a 13-year-old patient via headless compression screws by Medical Magnesium GmbH [122]. Postoperative clinical examinations showed excellent function, a rapid return to everyday activities including sports, and ultimately no pain. The postoperative MRI scans showed good healing and progressive biodegradation of the inserted implants [122]. In a study by Delsmann et al. [114], 29 patients (children and adolescents) that were treated with MAGNEZIX compression screws for fracture fixation, osteotomy, or osteochondral refixation were retrospectively analyzed by assessment of digital radiographs at the first and second follow-up visit 6 to 8 weeks and 12 to 24 weeks after surgery, respectively. Radiolucent zones were found to be a common but self-limiting phenomenon in the course of implant degradation, and neither implant failure nor impaired implant function was observed in any patient. However, the microstructural bone changes in direct vicinity of radiolucent zones remain unclear and require further study. In contrast, a retrospective case series of 18 patients, most of them undergoing shoulder surgery, by Haslhofer et al. [123] found a high complication rate (33.33%) due to material failure (4 patients) and infection (2 patients). The authors also observed a high percentage of radiolucency around MAGNEZIX CS screws, which however regressed over time and showed no clinical relevance.

Together, the formation of radiolucent zones around Mg-based implants appears to be a temporary occurrence during the resorption process, which should not be confused with implant loosening, infection, or osteolysis. Likewise, market-available second-generation implants seem to result in less prominent radiolucent zones, which might be the result from introduction of poly(ethylene oxide) (PEO)-treated surfaces that reduce initial degradation rates. However, further mechanistic insights are required to understand not only the osteoanabolic effect of Mg-based implants but also how Mg degradation products account for the occurrence of radiolucent zones. In this regard, one notable study reported the intriguing fact that the concentration of hydrogen in the gas cavities formed after subcutaneous Mg implantation in mice was low, challenging the scientific consensus that peri-implant cavities contain mainly H_2_ gas [93]. This suggests that hydrogen is rapidly exchanged after Mg corrosion and emphasizes the need for a deeper understanding of the mechanism behind the formation of gas cavities and the body's process for disposing of the H_2_ gas generated during Mg degradation.

Regarding further optimization of Mg-based implants, a slow degradation is associated with a lesser likelihood of the formation of temporary radiolucent zones. One approach is the addition of inorganic or organic coatings on the surface of Mg-based metals, being considered as an effective strategy to control the degradation rate of Mg-based implants [124–126]. Here, producing Mg-based implants with appropriate degradation characteristics is currently achieved by using alloying and surface modification techniques [12], most commonly by modification of the base material through consideration of suitable alloying systems with optimal amounts of alloying elements [127,128]. As stated above, the MAGNEZIX implant (Syntellix), which consists of a Mg alloy containing Mg, zirconium, and rare earth metals (MgYREZr), is one option for Mg-based implants and appears to have an adequate degradation rate for clinical use. [15,108–117]. Furthermore current in vivo and in vitro studies of the Mg alloy WE43 treated with a plasma electrolytic coating show that the coating significantly reduces the mass loss rate [120], representing an important step in further optimizing Mg-based implants. However, due to biocompatibility restrictions and mechanical performance reasons, the best corrosion performer may not always be the alloy of choice. In such cases, further modification of the material may be required. Forming of the base material or subsequent heat treatments may positively alter the microstructure and an associated degradation response [129,130]. To avoid an initial burst release of degradation byproducts by formation of the degradation layer right after implantation of the bare metal implant, surface treatments are usually applied to the Mg-based implant’s surfaces. Typical approaches consider PEO treatments that result in the formation of a degradation delaying MgO surface layer or treatments with fluorine to form a degradation delaying MgF_2_ surface layer [131,132]. The aforementioned approaches are usually combined to result in a slowly degrading implant with only little potential to result in appearance of radiolucent zones. It needs to be noted that, also in case of appearance of radiolucent zones, these have proven to be temporary and bone healing does not seem to be adversely influenced [114].

## Conclusion

Mg-based implants are a promising innovation and may represent the future direction in orthopedic surgery. A rising number of studies validate the effects of Mg-based implants on bone tissue healing, demonstrating excellent biocompatibility in connection with natural cortical bone, a physiological favorable Young’s modulus, biodegradation properties that result in avoidance of implant removal, and overall potent osteoinductive properties. Mg ions from Mg-based implants affect numerous and central biological pathways that control osteogenesis, osteoclastogenesis, angiogenesis, and inflammatory responses. Although a variety of biological effects of Mg-based implants are now well understood, further clinical and basic research is warranted to delineate specific aspects of Mg action in skeletal tissues. This includes the occurrence of radiolucent zones, which delay a more widespread clinical use of Mg-based implants in orthopedic trauma surgery. In this regard, surface modification strategies have evolved to further decrease the rate of degradation and may broaden the potential applications of Mg-based implants to treat challenging bone diseases, particularly in weight-bearing skeletal sites. Successful applications of WE43-based implants in orthopedic and trauma surgeries in the past years have already introduced this new material in the medical field.

## Materials and Methods

PubMed, Web of Science, and ScienceDirect databases were searched, and a manual search was performed using Google Scholar. Examples of key words used included Mg, Mg alloys, surface conversion coating, fracture, bone regeneration, bone remodeling, bone formation, bone resorption, radiolucent zones, angiogenesis, inflammation, osteoblasts, osteoclasts, stem cells, endothelial cells, immune cells, clinical trial, or a combination thereof. Titles and abstracts were read, followed by a second screening of the full text. Literature screening was performed independently by the 2 authors, and discrepancies were resolved by discussion or consultation with another author.
