# Supplementary material for: The Biological Effects of Magnesium-Based Implants on the Skeleton and Their Clinical Implications in Orthopedic Trauma Surgery
Source: Biomater Res. 2024 Dec 23;28:0122. doi: 10.34133/bmr.0122 (PMC11665827; doi:10.34133/bmr.0122)
Supplement: Supplementary 1 — Figs. S1 and S2 [file bmr.0122.f1.zip › Supplementary Materials.docx]

**Supplemental Figure 1.** Occurrence of radiolucent zones. CT scans of a patient with a distal humerus fracture (type Dubberley 2B) directly postoperatively (initial) and at 3 weeks follow-up. Evolving radiolucent zones in close vicinity of the implanted Mg-based screw (MAGNEZIX^®^, Syntellix) is indicated by red circles (left= axial view; right=frontal view).

**Supplemental Figure 2.** mm.X manufacturing. Sequence of processes that are required for the manufacture of mm.X magnesium-based implants. Manufacturing of mm.X products is conducted by meotec GmbH.
