# Supplementary figures and images for: The Biological Effects of Magnesium-Based Implants on the Skeleton and Their Clinical Implications in Orthopedic Trauma Surgery
Source: Biomater Res. 2024 Dec 23;28:0122. doi: 10.34133/bmr.0122 (PMC11665827; doi:10.34133/bmr.0122)

initial

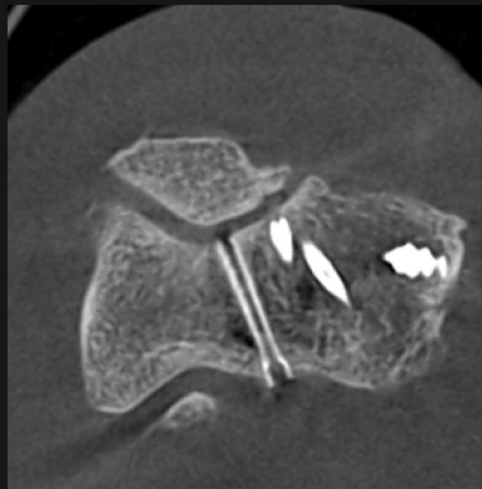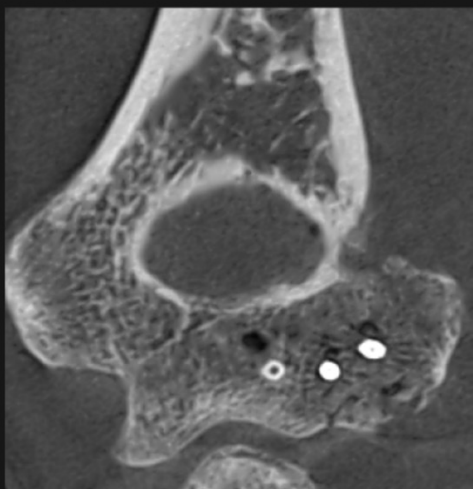

follow up 3 weeks

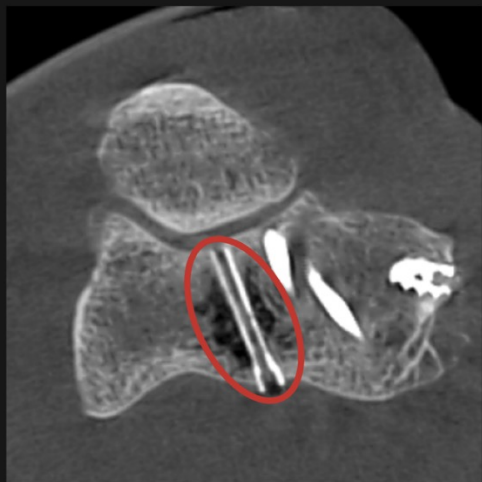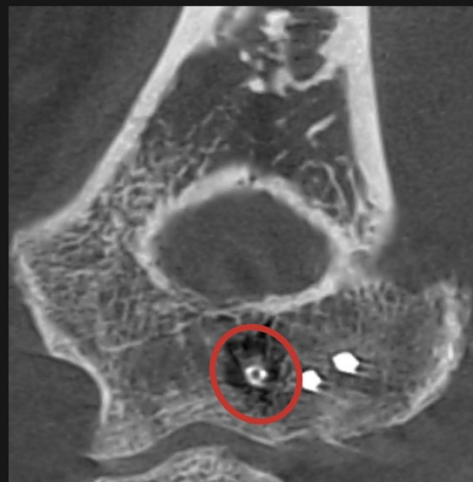

Supplement: Supplementary 1 — Figs. S1 and S2 [file bmr.0122.f1.zip › Figure S1.pdf]

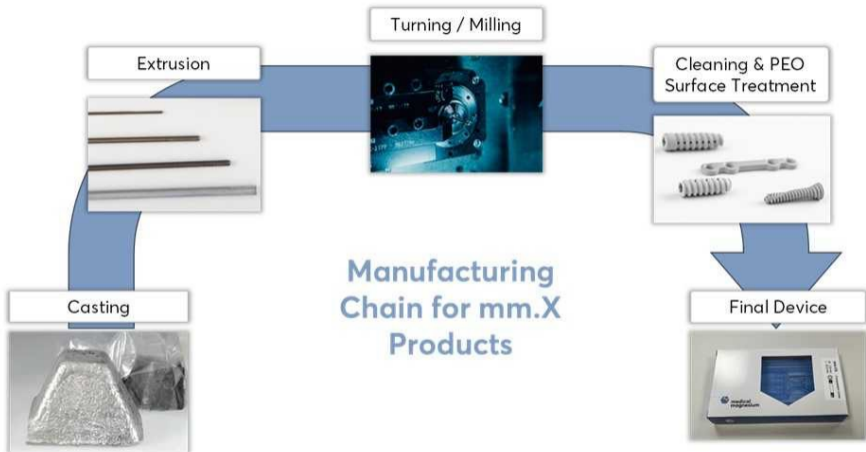

Supplement: Supplementary 1 — Figs. S1 and S2 [file bmr.0122.f1.zip › Figure S2.pdf]
